# An Ecological Momentary Assessment Protocol to Measure Stress, Socialization, and Other Contributors to Smoking Behaviors Among LGBTQ+ Adolescents: Multimethod Evaluation of Feasibility, Acceptability, and Appropriateness From the Puff Break Research Study

**DOI:** 10.2196/79957

**Published:** 2026-01-21

**Authors:** Linda Salgin, Daniel Kellogg, Irish Edusada, Andy C Lim, Amanda Velasquez, Jonathan Helm, Aaron J Blashill, Mark Myers, Hee-Jin Jun, Jerel P Calzo

**Affiliations:** 1 Joint Doctoral Program in Public Health, San Diego State University and University of California San Diego, 5500 Campanile Drive, San Diego, CA, 92182, United States, 1 8183247152; 2 Institute for Behavioral and Community Health, San Diego State University Research Foundation, San Diego State University, San Diego, CA, United States; 3 San Diego State University Research Foundation, San Diego State University, San Diego, CA, United States; 4 School of Public Health, San Diego State University, San Diego, CA, United States; 5 Department of Psychology, College of Sciences, San Diego State University, San Diego, CA, United States; 6 Joint Doctoral Program in Clinical Psychology, San Diego State University and University of California San Diego, San Diego, CA, United States; 7 VA San Diego Healthcare System Department of Psychiatry, University of California, San Diego, San Diego, CA, United States

**Keywords:** cigarette, feasibility studies, marijuana smoking, mobile apps, sexual and gender minorities, smoking, tobacco use cessation, vaping

## Abstract

**Background:**

Smartphone-based ecological momentary assessment (EMA) methods highlight the impact of minority stress and socialization (eg, discrimination and social support) on smoking behaviors in lesbian, gay, bisexual, transgender, queer, and other sexual and gender minority (LGBTQ+) adults; however, studies among LGBTQ+ adolescents are limited. The Puff Break EMA protocol was developed to address this gap.

**Objective:**

This study aims to report on the acceptability, feasibility, and appropriateness of the Puff Break EMA protocol.

**Methods:**

We utilized a multimethod design to evaluate the acceptability, feasibility, and appropriateness of the Puff Break EMA protocol. Participants who reported tobacco/nicotine or cannabis product use within the last 30 days engaged in a 2-week EMA trial, receiving 5 daily assessments measuring tobacco, nicotine, and cannabis use, stress and socialization, and product craving. Posttrial, participants completed a 15-minute exit survey and 60-minute semistructured exit interview. The exit survey used the 12-item Weiner acceptability, appropriateness, and feasibility measures and 6-item Mobile Application Rating Scale, app-specific subscale and also included 7 open-ended responses. The exit interview focused on a review of participants’ data to help understand smoking patterns and experiences with the Puff Break EMA protocol along with questions guided by the Reach, Effectiveness, Adoption, Implementation, and Maintenance framework to understand how a future EMA mobile intervention aimed at monitoring and reducing tobacco, nicotine, and cannabis product use could effectively be disseminated to, accessed by, and implemented with LGBTQ+ adolescents. Lastly, lessons learned were obtained through feedback and data collected throughout the study.

**Results:**

All 50 adolescents between the ages of 15‐19 (mean 17.82, SD 1.19) were enrolled in the study August 2023 and July 2024. Participants predominantly reported using vaporized tobacco and nicotine products (47/50, 94%), followed by cannabis products (39/50, 78%). The study sample was diverse regarding sexual orientation and gender identities with 32% (16/50) identifying as gay or lesbian, 32% (16/50) bisexual or pansexual, and 14% (7/50) transgender (neither transmasculine nor transfeminine). The median EMA response rate was 75% (~53 of 70 EMA surveys). Results indicated high feasibility (mean 4.43, SD 0.77), acceptability (mean 4.15, SD 0.83), and appropriateness (mean 4.46, SD 0.67) of the Puff Break EMA protocol. The Mobile Application Rating Scale app-specific subscale also indicated high acceptability and feasibility for the EMA method to increase knowledge, awareness, and intentions to monitor tobacco/nicotine use (mean 4.14, SD 1.01). Triangulated results from closed and open-ended survey responses identified 5 key themes related to feasibility, acceptability, and appropriateness. Participants highlighted the ease of the Puff Break EMA protocol, prompt survey reminders, and increased product use awareness. Key feedback from exit interviews included increased flexibility for survey timing, better response-option alignment, and appropriate only for populations interested in monitoring or reducing their product use.

**Conclusions:**

Findings indicate that using EMA methods to understand the impact of stress and socialization experiences on smoking behaviors in LGBTQ+ adolescents is feasible, appropriate, and acceptable.

## Introduction

According to the 2024 National Youth Tobacco Survey, 8.1% of all middle and high school students surveyed reported current use of tobacco products, with 2.8% of youth reporting combustible cigarette use and 5.9% of middle and 7.8% of high school students reporting vaping [1] Although rates of e-cigarette use or vaping declined from the previous year, pointing to the continued success of public health policy and prevention efforts, the data also found that 38.4% of students who vaped reported frequent use (>20 out of 30 d) and 26.3% reported daily use [1]. Furthermore, recent US national data from the Monitoring the Future study indicate that past-year cannabis use remains substantial among adolescents despite declines in recent years, with 26% of 12th graders, 16% of 10th graders, and 7% of 8th graders reporting past year use [2]. Adolescents who use e-cigarettes are also more likely to co-use cannabis products. Findings from the Population Assessment of Tobacco and Health study found that 51% of adolescents aged 12‐17 who ever used e-cigarettes also used cannabis in the last year [3]. Similar studies have found that adolescents who have ever used e-cigarettes had approximately 2.5 to 3.5 times higher odds of ever using cannabis products [3,4]. Taken together, early prevention of tobacco, nicotine, and cannabis use in all forms is critical. Such prevention efforts are particularly important among subpopulations who are at increased risk for tobacco, nicotine, and cannabis use.

Gender and sexual orientation disparities in tobacco and nicotine use emerge in adolescence and continue to persist into young adulthood, with lesbian, gay, bisexual, transgender, queer, and other sexual and gender minority (LGBTQ+) youth reporting earlier onset and elevated levels of tobacco and nicotine use relative to their cisgender- and heterosexual-identified peers [5-8]. LGBTQ+ youth are also more likely to engage in cannabis use than their cisgender and heterosexual peers [9]. Minority stress experiences (eg, bias-based victimization) and community socialization factors (eg, LGBTQ+ community norms regarding substance use) are unique, primary contributors to elevated risk for tobacco, nicotine, and cannabis use among LGBTQ+ youth [10-13]. Additionally, stress, interpersonal socialization factors, and tobacco, nicotine, and cannabis-related cravings are established influences on smoking and vaping behavior in the general adolescent population [14-19]. Research that delineates how such unique LGBTQ+ and general adolescent factors operate on a daily basis to shape tobacco, nicotine, and cannabis use can help inform prevention efforts. Despite the growth in research to examine the contributions of real-time psychological and contextual influences on substance use behaviors among LGBTQ+ adults [20-22], less research has advanced such assessment methods among LGBTQ+ adolescents. Ecological momentary assessment (EMA)—a technique for assessing the context, antecedents, and correlates of behavior in real-time—can be useful for understanding how daily factors affect tobacco, nicotine, and cannabis use among LGBTQ+ youth.

The Puff Break Research Study (Puff Break) used an EMA tool that includes validated and novel measures to capture the momentary associations between general stress, minority stress, socialization, craving, and other predictors of tobacco, nicotine, and cannabis product use among 50 LGBTQ+ adolescents, aged 14 to 19 years. The protocol was adapted from a prior EMA research protocol to examine smoking and vaping among LGBTQ+ adults. Puff Break was developed through an iterative design process including formative interviews from LGBTQ+ adolescents who engage in tobacco, nicotine, and cannabis use; key informant interviews with stakeholders, and subject matter experts in LGBTQ+ health, tobacco regulatory science, and EMA research. Feedback was obtained from a participatory planning group (PPG) consisting of public health professionals and representatives from nonprofit organizations focused on serving LGBTQ+ youth.

If successful at capturing antecedents and correlates of smoking behavior, the Puff Break EMA tool [23] has potential to inform real-time interventions among LGBTQ+ youth. The aim of this paper is to report on the feasibility, acceptability, and appropriateness of the Puff Break EMA protocol among LGBTQ+ adolescents via a multimethod evaluation along with key lessons learned.

## Methods

### Overview

Puff Break was a 2-week EMA trial where participants received 5 daily surveys evaluating tobacco/nicotine and cannabis use in addition to behavioral and contextual factors surrounding such use. Posttrial, participants completed an exit survey evaluating the feasibility, acceptability, and appropriateness of the Puff Break EMA protocol.

### Ethical Considerations

All study procedures were reviewed and approved by the San Diego State University Institutional Review Board (HS-2022‐0101). Participants provided informed consent at the start of the study during their onboarding. Due to the potential risk of “outing” LGBTQ+ youth and disclosing unknown youth-smoking behaviors to their parents and guardians, a waiver of parental consent for participants under the age of 18 was authorized by the SDSU Institutional Review Board. All data collected were deidentified for analysis. Identifiable information was securely stored on a San Diego State University password-protected server with only access to study personnel. Participants were compensated up to US $255 for participation in the study. Details of our compensation structure can be found in the Puff Break protocol paper [23].

### Study Design

This study used a multimethod design evaluating acceptability, feasibility, and appropriateness of the Puff Break EMA protocol through quantitative assessment (eg, completion rates, survey assessment with Likert scale ratings, and open-ended questions) and qualitative data (eg, semistructured interviews). The use of multimethods offers the opportunity for quantitative data to be further contextualized by qualitative open-ended responses, and the overall triangulation of results from different data sources provides a more comprehensive understanding of the results.

#### Participants and Recruitment

Participants for this study included LGBTQ+ adolescents who met the following inclusion criteria: (1) individuals who report current tobacco, nicotine, or cannabis product use (ie, any use in the past 30 d); (2) who are between the ages of 14 and 19 years; (3) who self-identify as LGBTQ+, and (4) who have daily access to a personal smartphone. A target sample size of 50 participants was selected to provide adequate power for future quantitative analysis as well as sufficient qualitative data to reach saturation of key ideas and themes. Participants were recruited from the greater San Diego County area between August 2023 and November 2024. We prioritized this region per the recommendations of our PPG and funding mechanism priorities. Multiple methods were used to recruit potential participants. Initial recruitment included distributing flyers, tabling at local youth and LGBTQ+ community events, and presenting to community and academic partners, and participant snowball sampling. All 7 flyers were developed by the Puff Break study team and were posted at a local school district, universities (eg, student union and LGBTQ+ resource center), and community settings (eg, library). A total of 8 presentations were conducted to community and academic partners along with 3 tabling events. Enrolled participants also provided referrals for individuals who may be eligible and interested in joining the study. As these efforts did not sufficiently engage youth younger than 18 years, we expanded recruitment through the use of social media. Using Meta (Facebook and Instagram), ad campaigns were iteratively created and configured to appeal to our target population. Advertisements were posted weekly and included 1 of our 7 flyers along with a short statement encouraging participation. The use of social media, along with adapting our protocol to include full remote procedures, increased our recruitment efforts. Further details of our recruitment procedures can be found in the Puff Break protocol paper [23].

#### Data Collection Procedures

Participants engaged in the 2-week Puff Break EMA protocol, receiving 5 daily surveys evaluating tobacco and nicotine use along with stress and socialization factors. Posttrial, participants engaged in an exit meeting which included a quantitative exit survey and qualitative exit interview further evaluating the acceptability and feasibility of the EMA protocol.

#### Quantitative Data Collection Procedures

Participants completed a 15-minute exit survey with open and closed-ended questions to assess acceptability, feasibility, and appropriateness, 3 key measurements of implementation outcomes, deemed indicative of an intervention’s success [23]. Acceptability was operationalized as the perception among LGBTQ+ adolescents that the Puff Break EMA protocol was appealing, favorable, or satisfactory. Feasibility was operationalized as the extent to which the Puff Break EMA protocol could be successfully implemented among the target population (ie, number of participants who met the recommended threshold of completing 80% of scheduled EMA assessments). Appropriateness was operationalized as participant ratings of the Puff Break EMA protocol as fitting, suitable, applicable, or a good match to assess smoking behaviors. Close-ended questions were adapted from the 12-item acceptability of intervention measure, intervention appropriateness measure, and feasibility of intervention measure by Weiner et al [24] with expectations to reach a threshold of agree or strongly agree from the Likert scales . Pearson correlation coefficients for the original study scales were 0.83 for acceptability, 0.87 for appropriateness, and 0.88 for feasibility, indicating acceptable test-retest reliability[24]. As the scale is meant to be pragmatic and used across diverse settings and audiences, no adaptations were made. We also assessed the perceived impact of an app on the user’s knowledge, attitudes, and intentions to change as well as the likelihood of actual change in the target health behavior via the validated 6-item Mobile Application Rating Scale (MARS) app-specific subscale [25,26]. The original MARS scale was found to have high internal consistency (α=.90) and interrater reliability, intraclass correlation coefficient (ICC=.79) [25]. Minor adaptations to questions included changing the word “improving” to “monitoring” and “increase/decrease” to “change.” All items were measured via a 5-point Likert scale (1=strongly disagree, to 5=strongly agree). A total of 7 open-ended questions were adapted from the study team’s adult EMA protocol which included, for example, the following questions: What did you like/dislike about the EMA method? What procedures were difficult to complete, confusing, or required additional support?

#### Qualitative Data Collection Procedures

A semistructured interview administered at the exit meeting further elucidated participants’ experience with the Puff Break EMA protocol. The interview guide had 15 scripted questions, with additional probes. The first half of the interview focused on a review of participants’ data to help understand smoking patterns and experiences with the EMA protocol. The second half of the interview was guided by the Reach, Effectiveness, Adoption, Implementation, and Maintenance framework [27] to understand how a future EMA mobile intervention aimed at monitoring and reducing tobacco, nicotine, and cannabis product use could effectively be disseminated to, accessed by, and implemented with LGBTQ+ adolescents. Interviews were recorded with participant consent and lasted approximately 60 minutes. Each participant received a US $25 gift card for completing the interview.

#### Lessons Learned

Lessons learned were obtained through iterative feedback from study staff, analysis of research participant qualitative data, and feedback from Puff Break PPG members consisting of 5 leaders within the local county health department and nonprofit organizations focused on serving LGBTQ+ youth to consult on their expertise related to the goals of the project.

### Data Analysis

We first assessed demographic characteristics of the sample population, from the screening survey at baseline, and then calculated descriptive statistics as seen in Table 1. To determine feasibility, we conducted a descriptive analysis of sample characteristics and compliance data (ie, number of participants who met the recommended threshold of completing 80% of scheduled EMA assessments) along with mean survey scores according to the feasibility of intervention measure [24]. We also conducted a content analysis of the open-response feedback from the exit survey regarding protocol feasibility. To determine acceptability, we similarly conducted a descriptive analysis of the mean survey scores according to the acceptability of intervention measure [24] along with a content analysis of open-ended response feedback from the exit survey. For additional context, we also conducted a descriptive analysis of the protocol’s appropriateness using the intervention appropriateness measure [24], content analysis of open-ended response feedback from the exit survey, along with analyzing mean survey scores from the MARS app-specific subscale [25,26] to understand the protocols perceived impact on users’ knowledge, attitudes, and intentions to use an EMA protocol like Puff Break to monitor their tobacco, nicotine, and cannabis product use. Participants were retained in all analyses even if they had missing responses for individual items.

**Table 1.** Demographic characteristics of the 50 enrolled participants in the Puff Break pilot study, August 2023 to November 2024.

Characteristic	Count, n (%)
Participants (enrolled)	50 (100)
Age^a^ (years)	
14	0 (0)
15	4 (8)
16	3 (6)
17	7 (14)
18	20 (40)
19	16 (32)
Sex assigned at birth	
Female	42 (84)
Male	8 (16)
Gender identity	
Woman	20 (40)
Man	11 (22)
Nonbinary/genderfluid	9 (18)
Multiple gender identities	10 (20)
Transgender status	
Transmasculine	9 (18)
Transfeminine	0 (0)
Transgender, neither transmasculine nor transfeminine	7 (14)
No	31 (62)
Questioning	2 (4)
Not reported	1 (2)
Sexual orientation	
Lesbian/gay/queer	16 (32)
Bisexual/pansexual	16 (32)
Other written-in response	1 (2)
Multiple sexual orientation identities	17 (34)
Ethnicity	
Non-Hispanic or Latino/a/x	31 (62)
Hispanic or Latino/a/x	19 (38)
Race^b^	
White	27 (54)
Non-white	8 (16)
Multiracial or multiethnic	14 (28)
Not reported	1 (2)
Past 30 day tobacco/nicotine product use (any)^c^	
Combustible products	27 (54)
Smokeless products	8 (16)
Vaporized products	47 (94)
Cannabis products	39 (78)
Other products	2 (4)
Combustible or vaporized product use: 5 times or more in a typical day^c^	
Combustible products	0 (0)
Vaporized products	33 (66)

a Age: mean (SD; range)=17.82 (1.19; 15-19) years.

b Race was a 9-category item that was collapsed to 3 levels due to small cell count: White (included White and Jewish); non-White (included Black/African American, Native American/Alaskan Native, Asian, Southeast Asian); Multiracial or multiethnic (included respondents who selected more than one category, eg, White and Black/African American).

c Responses are not mutually exclusive.

Data triangulation was used by comparing themes from open-ended survey responses with those derived from participant interviews. Interview transcriptions were obtained from Zoom’s (Zoom Communications, Inc) cloud recording function. Transcripts were cleaned, memoed, and extracted into an Excel sheet to identify crosscutting trends in the data, leading to the generation of a preliminary codebook [28]. Subsequent analysis was conducted using Nvivo 14.23.4 (Lumivero, Inc) [29]. The analysis team simultaneously reviewed, discussed, and coded 20% of the transcripts, upon which the codebook was refined. Once the team was comfortable with the codebook, the lead author and research assistant independently coded another 20% of the transcripts to establish an interrater agreement of at least 80%. Discrepancies were discussed and resolved between the coders. Once interrater agreement was achieved, the remaining transcripts were independently coded. Once all transcripts were coded, the analysis team engaged in multiple rounds of dialogue to identify key themes and patterns across the data.

## Results

### Demographics

**Figure 1.**
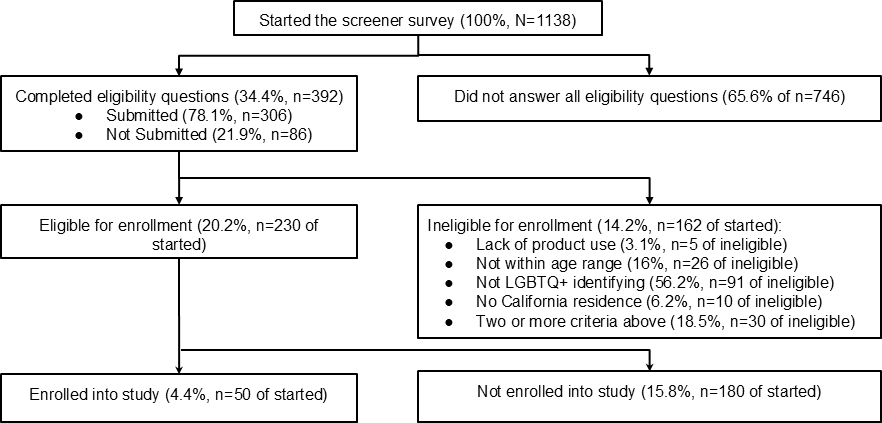
CONSORT (Consolidated Standards of Reporting Trials) diagram displaying enrollment of participants in the Puff Break study based on youth who started the screener survey between August 2023 and November 2024. Those who completed eligibility questions but did not submit the screener were still contacted to join the study. Reasons for not enrolling included: not responsive to staff messages, did not attend onboarding meetings, and no longer interested. LGBTQ+: lesbian, gay, bisexual, transgender, queer, and other sexual and gender minorities.

Recruitment efforts reached a total of 1138 potentially eligible participants, of which 306 completed the screener survey. Of these respondents, 230 were deemed eligible to participate based on our inclusion criteria. Ultimately, 50 participants were enrolled and completed the study between August 2023 and November 2024. The screener survey results are displayed in Figure 1.

As displayed in Table 1, participants’ ages ranged from 15 to 19 years (mean 17.82, SD 1.19) with 8% (4/50) aged 15, 6% (3/50) aged 16, 14% (7/50) aged 17, and the majority (36/50, 72%) between 18 and 19 years. The majority of participants (42/50, 84%) identified as female sex at birth; 18% (9/50) of the study sample identified as transmasculine, and 14% (7/50) identified as transgender (neither transmasculine nor transfeminine). For sexual orientation, 32% (16/50) of participants identified as lesbian/gay/queer, 32% (16/50) identified as bisexual/pansexual, and 34% (17/50) identified as having multiple sexual orientation identities. Approximately 40% (19/50) of the participants identified as Hispanic/Latino/a/x, 54% (27/50) identified as White, and 28% (14/50) identified as multiracial. Participants reported using a range of tobacco and nicotine products in the last 30 days, including tobacco: combustible cigarettes (27/50, 54%), smokeless tobacco (8/50, 16%), vaporized nicotine (47/50, 94%), cannabis (39/50, 78%), and other types of products (2/50, 4%). While no participants reported using combustible products more than 5 times in a typical day, 66% (33/50) of participants reported using vaporized products at least 5 times in a typical day.

### Feasibility, Acceptability, and Appropriateness of the Puff Break EMA Protocol

#### Feasibility

We were able to enroll 50 participants, with 100% of participants completing the study entirely (onboarding to exit). Participants were sent 5 surveys a day during the 2-week EMA trial (maximum of 70 surveys per participant). Participants’ response rate varied, ranging from 12.9% (~9 of 70 EMA surveys) to 97.1% (~68 of 70 EMA surveys), with a median response rate of 75% (~53 of 70 EMA surveys). Additionally, 22 out of 50 participants (44%) met the 80% threshold for completing EMA surveys (56 of 70 EMA surveys). Participants were typically involved in the study for 16 days from onboarding to exit interview. Regarding subjective ratings, the Puff Break EMA protocol was given high scores on the feasibility of intervention measure (mean 4.43, SD 0.77).

Triangulated results from the open-ended responses and interview elucidated 1 overarching theme of *usability* with multiple subcomponents related to *timing and access*. Related to timing, participants highlighted how the structured timing of the EMA surveys made it challenging to complete their assessments. For instance, 1 participant said,

*I mean I did take the 9 AM surveys a few times, but I also just never really woke up early enough to do so.*

[19 y, Asian, nonbinary, and multiple sexual orientation identities]

while others also indicated:

*The only thing that I did struggle with was just like the time cause you know how it has, like specific times. Yeah, sometimes I’m like at work, or I’m just sleeping, and I don’t, I don’t see them.*

[18 y, multiracial, woman, and pansexual]

and

*I think just like a more open time period, because I’d click on it like right at like 3 o’clock or like 6 o’clock, and sometimes I couldn’t do it and like I would have work at like 3:30, or like 4, and then it wouldn’t show up until, like a random time like 3:27, and I’d be like could have done this like 27 min ago. Now, I can’t do it.*

[17 y, White, woman, and bisexual]

Related to access, the analysis indicated that approximately 10% (5/50) of participants expressed challenges with app glitches and how that affected their ability to input their information, with participants stating,

*The app can be very glitchy and unreliable, such as not allowing the users to open the survey when it’s the required time to take it.*

[17 y, White, woman, and bisexual]

and

*It like would crash a lot, and just like in the middle of it, I’d be like great. I had to do this all over again.*

[18 y, Hispanic, transmasculine, and gay]

Notably, 1 participant brought to light challenges with accessing their cell phone due to parental controls which contributed to their low response rates for evening surveys:

*Either your parents give you free rein and don’t care about what you do with your technology or your parents like mine, where they check your phone every week and like you’re not allowed to have your phone with you overnight. And I have like very supervised technology usage.*

[17 y, nonbinary/transmasculine, and gay]

Despite these challenges, 24% of participants expressed their overall satisfaction of being part of the study. For example, participants stated,

*It was interesting, my first experience with documenting or keeping track of how I use substances and in regards to my emotions as well. It will be interesting to see what results might come out after this study is over.*

[15 y, White, transmasculine, and multiple sexual orientation identities]

Similarly, another participant also expressed that they enjoyed the experience:

*This was actually kind of fun, I liked that it was a scheduled thing and it put me into a cycle, a routine of sorts.*

[18 y, White, woman, and gay/queer]

Lastly, 50% (25/50) of participants highlighted that the Puff Break EMA protocol was easy to use, with 1 participant stating,

*I really like how like it didn’t take that much time out of my day, and it was something that I could just easily get done throughout the day.*

[17 y, Hispanic/White, woman, and multiple sexualities]

#### Acceptability

The Puff Break EMA protocol received high acceptability ratings via the acceptability of intervention measure (mean 4.15, SD 0.83). Triangulated results from the open-ended responses and interview elucidated 2 key themes of (1) *app features* and (2) *product use awareness*. Within *app features*, participants found the notifications to be beneficial, allowing them to stay on track with their EMA surveys. For example, 1 participant stated,

*Yeah, I think like I was just thinking of the how the survey sent me notifications, and like that made it easier to remember.*

[15 y, White, transmasculine, and multiple sexualities]

However, 1 participant indicated how the app felt very “assignments-y” coupled with the notification’s wording of “it’s time for your assessment” made them not want to complete surveys:

*It just kind of feels like very assignments-y.I think that the way the notifications are sent, like the words that are used, are kind of makes it harder, because, like they’re like, “it’s time for your assessment.” And if they didn’t say that, if they had like another wording, I think it- I’d be more inclined to at times.*

[19 y, Hispanic/Black, nonbinary, and bisexual/queer]

Acceptability was also seen through the theme of *product use awareness* where 37% of participants indicated how tracking their product use within the app, and the data visualizations viewed at the start of the exit interview, opened their eyes to their product use and made them reflect on what they could do to reduce use. As shared by 2 participants,

*I really liked like looking through like my stats right now [at the exit interview], like the graphs like my like, 2 week average like that was interesting. And I was like, Wow, like, especially for cannabis use like that’s crazy.*

[19 y, Hispanic/White, non-binary, and queer]

and

*I do think like the whole like tracking. How many times I’ve like taking hits. did help a lot like recognize my usage.*

[19 y, Asian, non-binary, and bisexual/queer]

This finding was further emphasized by another participant:

*I feel like how you’re showing you right now. It just kind of like puts it all into perspective, like what you’re doing and learn like you can think by how I am like oh, like can I come up with like a different habit to do it at these times. And like for me, I’m looking at. I’m like, okay, it’s at like 6 PM or [9 PM] at night, [going to gym] or something instead of just like smoking.*

[19 y, White, woman, and bisexual]

#### Appropriateness

The intervention appropriateness measure indicated that the Puff Break EMA protocol was viewed as appropriate among LGBTQ+ youth (mean 4.46, SD 0.67). The MARS app-specific subscale, which aimed to specifically evaluate the quality of mobile health apps, also indicated high appropriateness (mean 4.14, SD 1.01). Results indicated that the Puff Break EMA protocol could increase participants’ awareness, knowledge, attitudes, and intentions to monitor tobacco and nicotine as well as seeking help for or promoting a change in their tobacco and nicotine use behavior.

Triangulated results from the open-ended responses and interview elucidated three overarching themes of (1) *motivations to monitor or reduce tobacco use*, (2) *product use awareness*, and (3) *product-response option alignment*. Within *motivations to monitor or reduce tobacco use,* there was general consensus that the Puff Break EMA protocol and any future iterations of the study would only be appealing to individuals who were already interested in monitoring, reducing, or eliminating their tobacco and nicotine use as exemplified by feedback shared by the following participants:

*If I had been shown the app like that at the beginning, like when I first hit a vape like in the school bathroom freshman year, I would have been like, I don’t care. Like I don’t need that, whatever, like who cares, like I’m just having fun.*

[18 y, Hispanic/White, transmasculine, and bisexual]

*This research study kind of helped me since the app allowed me to track it But if the person doesn’t have the motivation to like Stop, there’s no like, that’s the only point of the app.*

[18 y, Asian, woman, and bisexual]

Regarding *product use awareness*, results elucidated how participating in the study made participants more aware of their general product use and how it related to their stress, mood, or other aspects of their lives. One participant stated,

*I was able to record my momentary moods, it was interesting to see how this corresponded with nicotine usage.*

[17 y, White, woman, and gay/queer]

while another similarly indicated,

*It made me consider a lot about my cravings and usage and how it correlated with my stress levels.*

[19 y, Asian, non-binary, and bisexual/queer]

Notably, while some participants discussed how participating in the EMA trial led to a decrease in their product use, 2 participants indicated how being in the study had an unintentional effect of increasing their cravings, stating,

*I think that’s what really pushed me to use less was when I was inputting those numbers. Like, if I went a period where I used seven times at once, then the next period I’d be fine with not even using it at all, just because I had seen how much I used it in such a short period of time.*

[18 y, Hispanic/White, transmasculine, and bisexual]

and

*...the [survey], like hella increased like my cravings every time I opened it, even just seeing the word nicotine seeing vaporized like when you’re trying to quit like seeing it is really rough.*

[19 y, White, man, and gay]

Lastly, in regard to the theme of *response option wording,* participants mentioned their appreciation for the EMA questions not being too invasive or triggering. Additionally, they felt as if the questions being asked were appropriately worded. A participant claimed,

*I feel EMA has a great system in keeping reasonable data without being invasive of noting common discomfort that may occur in LGBTQIA+ young adults.*

[19 y, White, woman, and bisexual]

However, 12% of the participants provided feedback highlighting that questions were either too narrow, honing in on specific LGBTQ+ stressors, or too broad, limiting their ability to dive deeper into their reason for using tobacco or nicotine. For instance, participants stated,

*I didn’t feel like it asked more nitty-gritty questions like “why did you start smoking?” or “what drives you to smoke?” in the first place.*

[19 y, Hispanic/White, nonbinary, and pansexual]

as well as

*I think that it would be helpful to identify the reasons for the stress score whether is was family, peers, occupation, or school.*

[18 y, White/Black, nonbinary, and queer/pansexual]

and

*I feel like there were not enough general “I am stressed” indicators because it was all about LGBT issues and not if I had a test earlier in the day or something.*

[17 y, White, genderqueer, and bisexual/queer]

Lastly, the *product-response option alignment* theme also alluded to challenges with the way in which our EMA prompts were worded. When showing participants their own data, many reported not recalling using “other” or “smokeless products.” Many participants confused smokeless products with other products. Some examples are mentioned below:

*I’m almost completely certain I missed clicked on every time I put smokeless. I have not used a single smokeless product.*

[17 y, Black/Asian, nonbinary/transmasculine, and demisexual]

*so I didn’t smoke any cannabis. So I don’t think I put that on the survey. Maybe it was an accident a few times or something. When I took the surveys I was pretty careful about not putting wrong [or] anything, so I’m just confused.*

[17 y, White, man, and bisexual]

*Or for- is it smokeless or other I’m speaking about? I guess maybe I confused like Smokeless and Other, and I might have like Cause like there’s like the like, not the patches, but like the gum ones, like the ones you put in your mouth. I might have like put those to confused them at one point, when I was doing the survey*

[19 y, Hispanic/Black, woman/nonbinary, bisexual/queer]

#### Lessons Learned

The study team learned several lessons when administering the protocol amongst their sample. Lessons included the need to validate EMA survey responses about product use, the inclusion of more comprehensive measurement of cannabis product use, the establishment of regular data documentation and inventorying, prompt technical assistance and communication about survey technology and schedules, and the verification of screener survey responses and participant IDs. Table 2 summarizes the lessons learned throughout the duration of the Puff Break EMA protocol and recommendations from the research team for future studies that used EMA methodology.

**Table 2.** Lessons learned and recommendations for future EMA research.

Study components and lessons learned	Recommendations for future EMA^a^ research
Acceptability	
Participants had mixed feedback regarding the default, fixed features of the EMA survey platform that the research team chose for Puff Break.	EMA researchers should thoroughly search and test the different EMA survey platforms that are available to ensure that the most appealing platform is chosen for their participants.
Feasibility	
EMA research participants can experience EMA tool issues at any point during the study and may need additional accommodations to their survey schedule to participate as much as possible.	Encourage EMA research participants to contact research staff as soon as they experience technical issues and train staff to respond promptly within the hours of the survey delivery time windows. Use a user-based survey schedule and allow the research participants to tailor their survey delivery time windows (such as an hour earlier or later) before their survey schedule is configured. Schedule SMS text messaging or Zoom check-ins with participants two days after the participant begins their EMA trial participation to catch technological or scheduling challenges before they severely impact response rates. Schedule another SMS text messaging or Zoom check-in halfway through the trial as well.
Appropriateness	
Product use responses needed to be validated by the participants during the exit interview as participants claimed to not use certain products that were shown in their survey responses.	Include data validations at the beginning of exit interviews, such as “we recorded responses for these types of products. Can you confirm whether or not you used each of these?” When training participants on how to use the EMA tool, instruct them to answer survey questions carefully to avoid misclicks. If possible, enable a backspace button for participants to edit responses before submitting their survey. Ensure that the definitions of product types are clear to the participants before they begin answering surveys, such as the differences between “smokeless” and “cannabis.” If the ability to customize the size of survey response text, or the distance between survey responses, do so to ensure responses are easy to read from an arm’s length and reasonably spaced between each other.
More cannabis product-related measures could have been used as sole cannabis use and cannabis co-use with tobacco and nicotine products was much more prevalent in the sample than anticipated.	Tobacco and nicotine product use measures should be adapted to include cannabis product use to reliably compare and contrast tobacco and nicotine, and cannabis product data.
Logistical (screening process)	
Online screener surveys are at risk for being flooded by bot-generated responses or ineligible persons who submitted disingenuous survey responses.	Develop a brief data integrity protocol to flag bot-generated or disingenuous survey responses. The Puff Break protocol instructed staff to flag responses that used formulaic email addresses, rapid screener survey completion times, data discrepancies within survey responses, or IP addresses outside of the United States. Implement ID verification before the participant enrolls in the study, using best judgment if the participant goes by a different name or gender identity. If on Zoom, require participants to use their camera and microphone. Leverage in-person community networks for recruitment to reduce the risk of encountering bot or disingenuous screener survey responses via online recruitment methods.
Logistical (EMA trial)	
Multiple, robust codebooks and data cleaning protocols were needed to handle myriad formatting challenges that arose throughout the data download and merging process, especially with unanticipated updates to the EMA host platform.	Regularly export and assess data formatting between survey participants to catch any changes made to the survey output and avoid challenges when merging participant datasets. Document edits to EMA surveys, datasets, codebooks, and protocols as they are being made to avoid duplicating work at a later date.

a EMA: ecological momentory assessment.

## Discussion

### Principal Results and Comparison With Prior Work

The purpose of this study is to use multimethod design to evaluate the acceptability, feasibility, and appropriateness of Puff Break, an EMA protocol measuring stress, socialization, and smoking behaviors among LGBTQ+ adolescents. Importantly, this study addresses a gap in the literature regarding the use of EMA methods and highlights the impact of minority stress and socialization (eg, discrimination and social support) on smoking behaviors in LGBTQ+ adolescents. Findings from 50 participants indicate that using EMA to understand the impact of stress and socialization experiences on smoking behaviors in LGBTQ+ adolescents is feasible, acceptable, and appropriate among LGBTQ+ adolescents aged 15-19 years. In particular, the study was able to recruit the intended sample size and had a 100% study completion rate among participants. Participants were prompted to complete 5 EMA assessments daily for 2 weeks, which resulted in a median response rate of 75%. The number of EMA assessments and duration of the protocol were determined by prior EMA research on substance use among adolescents and adults which ranged from 2 to 4 weeks [20,21,28]; (with 1 study highlighting the feasibility of using a 2-week EMA window among middle and high school students [30]), along our formative data collected in Phase 1 of the study which recommended this schedule as most feasible [23]. Our response rate was slightly lower than studies that had longer EMA trial periods of 4 to 12 weeks [31-33], which may be primarily attributed to design features (eg, survey timing) or technical challenges with the EMA tool (eg, app glitches) rather than the potential burden of completing 5 daily surveys. Some participants in our study repeatedly reported challenges with completing surveys due to work, school, and sleep schedules, with 1 participant indicating parental controls on cell phone usage as a barrier to survey completion. While some participants were generally less responsive, qualitative data suggested an overall positive experience and interest in the study, indicating that response rates could improve if these barriers are effectively mitigated. Additionally, in line with existing research on EMA and mobile interventions, the Puff Break study evaluated feasibility and acceptability in regards to enrollment completion rates [31-33]. However, this study extends beyond these measures by including appropriateness, including multimethod assessment to further elucidate the feasibility, acceptability, and appropriateness of the Puff Break EMA protocol.

Triangulated results from closed and open-ended survey responses as well as semistructured interviews indicated high scores for feasibility, acceptability, and appropriateness (range 4.15 to 4.46) and identified 5 key themes of (i) usability; (ii) product use awareness; (iii) app features; (iv) motivations to monitor or reduce tobacco use; and (v) product-response option alignment. These results highlight that the Puff Break EMA protocol is implementable among LGBTQ+ adolescents. Qualitative results highlight that the Puff Break EMA protocol was easy and simple to use with EMA surveys being straightforward and requiring minimal time for completion. The notifications and survey delivery, along with the simplicity and discretion of the EMA questions, were appealing to participants. Lastly, the inclusion of visualizations in the exit survey, along with tracking a wide variety of evidence-based predictors of tobacco, nicotine, and cannabis use, increased participants’ awareness of their product use and offered opportunities for reflection. Notably, we found that while participants believed the Puff Break EMA protocol was appropriate for both LGBTQ+ and non-LGBTQ+ adolescents, it may be most appealing to individuals with an existing intention to monitor or quit tobacco, nicotine, or cannabis use. This is consistent with general public health theories and other tobacco cessation interventions where individual intention is a key motivator to behavior change [34,35]. Further research is needed to extrapolate on the additional measures that elucidated the feasibility and acceptability of the Puff Break EMA protocol, as well as the appropriateness of EMA research to address tobacco, nicotine, and cannabis use among adolescents, regardless of gender identity and sexual orientation.

### Strengths and Limitations

The Puff Break EMA protocol has various strengths. First, the study recruited a diverse sample of LGBTQ+ adolescents across several racial and ethnic identities, gender identities, and sexual orientations. Though we were limited in participants who identified as transfeminine or assigned male sex at birth, the overall diversity allowed us to obtain varying lived experiences and unique perspectives in this pilot dataset. Second, we aimed to use a multitude of validated measures for tobacco and nicotine product use, stress, minority stress, socialization, craving, and mood. When curating measures for the EMA surveys and supporting assessments (eg, baseline and exit surveys), we used scales that were created, previously adapted for, or validated among adolescents such as the Sexual Minority Adolescent Stress Inventory [36] and Positive and Negative Affect Schedule [37]. When not available, we used validated instruments used extensively in prior research with adolescents, such as the Timeline Follow-Back instrument [38]. When needed, minor adaptations to validated measures were made to fit the context of our study. Measure adaptations were informed by community service providers, LGBTQ+ adolescents who smoke, and academic partners, which not only increased the quality of our measurement but also helped to develop an EMA protocol that is responsive to LGBTQ+ adolescents. Details of our measures and adaptations can be found in the Puff Break protocol [23]. Lastly, the use of multiple methods (eg, qualitative and quantitative) to gather robust feedback on the protocol led to iterative refinements that are likely contributing to the protocol’s high acceptability, feasibility, and appropriateness scores and general excellence across qualitative feedback.

Nevertheless, the study is not without limitations. First, our study may not be generalizable to the larger LGBTQ+ populations outside of San Diego County. Second, although it was straightforward to recruit LGBTQ+ aged 18 to 19 years, recruiting adolescents younger than 18 was challenging. Engaging the PPG members in direct recruitment assistance increased the recruitment of younger participants, but none aged under 15 years were enrolled with this additional support. We believe that difficulties recruiting participants under the age of 18 in our study, specifically those 14 to 16 years, are attributable to the lower rates of overall tobacco and nicotine product use among younger adolescents, regardless of sexual orientation. For instance, while 7.8% of high school students reported using e-cigarettes within the last 30 days, the percentage is halved (3.5%) for middle school students [39]; LGBTQ+ youth would be a small subset of this population (ie, some subset of the total 3.5%), causing the potential recruitment pool to be small.

Despite formative work to identify an acceptable EMA platform, a third limitation of the study concerned the technical difficulties that occurred during the EMA trial, such as participants not receiving planned push notifications to take their surveys, not being able to open surveys when survey windows were open, and the inability to prepopulate survey responses prepopulated from previous surveys. All of these unanticipated factors could have affected our response rates. Such issues could have been addressed by selecting an EMA operating system that was more reliable or that offered such design features. Participants were given instructions on soft resets, as well as encouragement to contact research staff to troubleshoot difficulties. Lastly, we acknowledge the potential for selection and participation bias in our sample. It is possible that enrolled participants in this study were already interested in decreasing or quitting their tobacco, nicotine, and cannabis use via a mobile tool, resulting in potential selection bias and limiting our understanding of feasibility, acceptability, and appropriateness among LGBTQ+ adolescents in precontemplative stages of tobacco, nicotine, or cannabis cessation. Our primary recruitment strategies (flyers, social events, and social media) and requirement of smartphone access may have preferentially attracted adolescents who were already more engaged or resourced, which could introduce selection bias. However, data from the Pew Research Center highlights that among US adolescents aged 13 to 17 years, even those experiencing financial hardship (eg, annual household income less than $35,000), 89% have access to a smartphone and 51% use the internet *almost constantly* [40], thus suggesting that smartphone access may not pose a substantial barrier to youth participation. In addition, we bolstered our recruitment efforts with additional strategies, including recruitment from drop-in centers and referrals from enrolled participants, which may have increased the diversity of youth included in the research. Taken together, our recruitment approach strove to balance feasibility with inclusivity.

### Conclusion

Despite the limitations identified, the Puff Break protocol demonstrated high feasibility, acceptability, and appropriateness. The Puff Break EMA protocol [23] has potential for further development to inform real-time tobacco, nicotine, and cannabis interventions among LGBTQ+ youth. Participants reported high acceptability, highlighted the app’s simplicity, and noted a perceived increase in awareness of their own product use behavior. Additionally, participants reported that the Puff Break EMA protocol is appropriate for monitoring tobacco, nicotine, and cannabis use behaviors and has potential for adaptation as an interventive tool. These findings, plus the lessons learned, indicate the promise for EMA methodology to be used in tobacco, nicotine, and cannabis research, and real-time prevention efforts among LGBTQ+ adolescents.

## Supplementary material

10.2196/79957 Checklist 1 STROBE checklist.

## Abbreviations:

EMA: ecological momentary assessment
LGBTQ+: lesbian, gay, bisexual, transgender, queer, and other sexual and gender minorities
MARS: Mobile Application Rating Scale
PPG: Participatory Planning Group
